# Short-term effects of Kinesio taping combined with cervical muscles multi-angle isometric training in patients with cervical spondylosis

**DOI:** 10.1186/s12891-023-06154-x

**Published:** 2023-01-18

**Authors:** Jian Xiong, Zhe Zhang, Zhichao Zhang, Yan Ma, Zuhong Li, Yongjin Chen, Qi Liu, Weijing Liao

**Affiliations:** 1 Department of Physical Medicine and Rehabilitation, Zhongnan Hospital of Wuhan University, Wuhan, 430071 China; 2 Department of Physical Medicine and Rehabilitation, Wuhan Hospital of Traditional Chinese and Western Medicine, Wuhan, 430030 China; 3 Department of Physical Medicine and Rehabilitation, Ezhou Central Hospital, Ezhou, 436000 China

**Keywords:** Cervical spondylosis, Isometric, Kinesio taping, Multi-angle, Resistance training, Stiffness

## Abstract

**Objective:**

The purpose of this study was to investigate the efficacy of Kinesio taping (KT) combined with multi-angle isometric resistance training for cervical spondylosis.

**Methods:**

Sixty-one patients were divided into two groups by random number table method. Both groups were given multi-angle isometric training, the patients in the observation group were supplemented with Kinesio taping. Before and after treatment, the symptoms of cervical spine function were evaluated in two groups by visual analogue scale (VAS), cervical dysfunction index (NDI), cervical range of motion and muscle stiffness.

**Results:**

After 3 weeks of treatment, VAS, NDI scores and the cervical range of motion were significantly better than before (*P* < 0.05). The range of anterior flexion and extension was significantly larger than the control group (*P* < 0.05), but the range of other motions were not certain. The muscle stiffness in KT group were significantly lower than the control group.

**Conclusion:**

Kinesio taping combined with multi-angle isometric resistance training can further alleviate the clinical symptoms and correct the neck abnormal posture. But its effects on the range of cervical motion remain uncertain.

## Background

Neck type cervical spondylopathy (NTCS), a type of cervical spondylosis, is characterized by pain in the neck and shoulder, limited range of movement, and abnormal physiological curvature. In recent years, due to the growing prevalence of poor posture caused by the overuse of Internet, the incidence of NTCS is increasing, especially in students. Therefore, it had a significant impact on daily life (including work and study) of patients with NTCS.

The most common treatment for the pain syndrome in the course of NTCS is physiotherapy, which include manual therapy and Kinesio taping(KT) [1]. Previous studies have confirmed the efficacy of manual therapy and Kinesio taping (KT) in alleviating symptoms of NTCS by altering parafunctional behaviors [2, 3]. Further studies found that the key to perform efficient manual therapy or KT was to localize and release those trigger points.

Trigger points can be localized by palpation examination with the manifestation of nodules at a size of granule. It was formed by muscle fibers with increased tension, which could interfere with muscle movement patterns and weaken muscle strength. The discomfort could be alleviated by releasing trigger points [4, 5].

(KT) has been widely used for treating various injuries [6–9] and attracted much attention in recent years. It can normalize muscle function, increase microcirculation, relieve pain, and support the work of joint [10–13] by applying specific tape on patients’ skin. Researchers have proposed it may reduce lymphatic and venous edema, but its effect in muscle disease and vascular condition was poorly reported. The exact mechanism of KT remains unclear. However, it was reported that KT may have short-term effects on muscle activation and support proprioceptive information [14, 15]. In this study, we investigated the effectiveness of Kinesio taping (KT) combined with multi-angle isometric resistance training on the pain and movement function in patients with NTCS.

### Methods and participants

#### Participants

Seventy patients with NTCS in the Department of Neurology and Rehabilitation in our hospital were recruited between March 2016 and December 2019. The assessors and statisticians were unaware of the group allocation throughout the study period. All the researchers received training to ensure strict adherence to the study protocol.

**Fig. 1:**
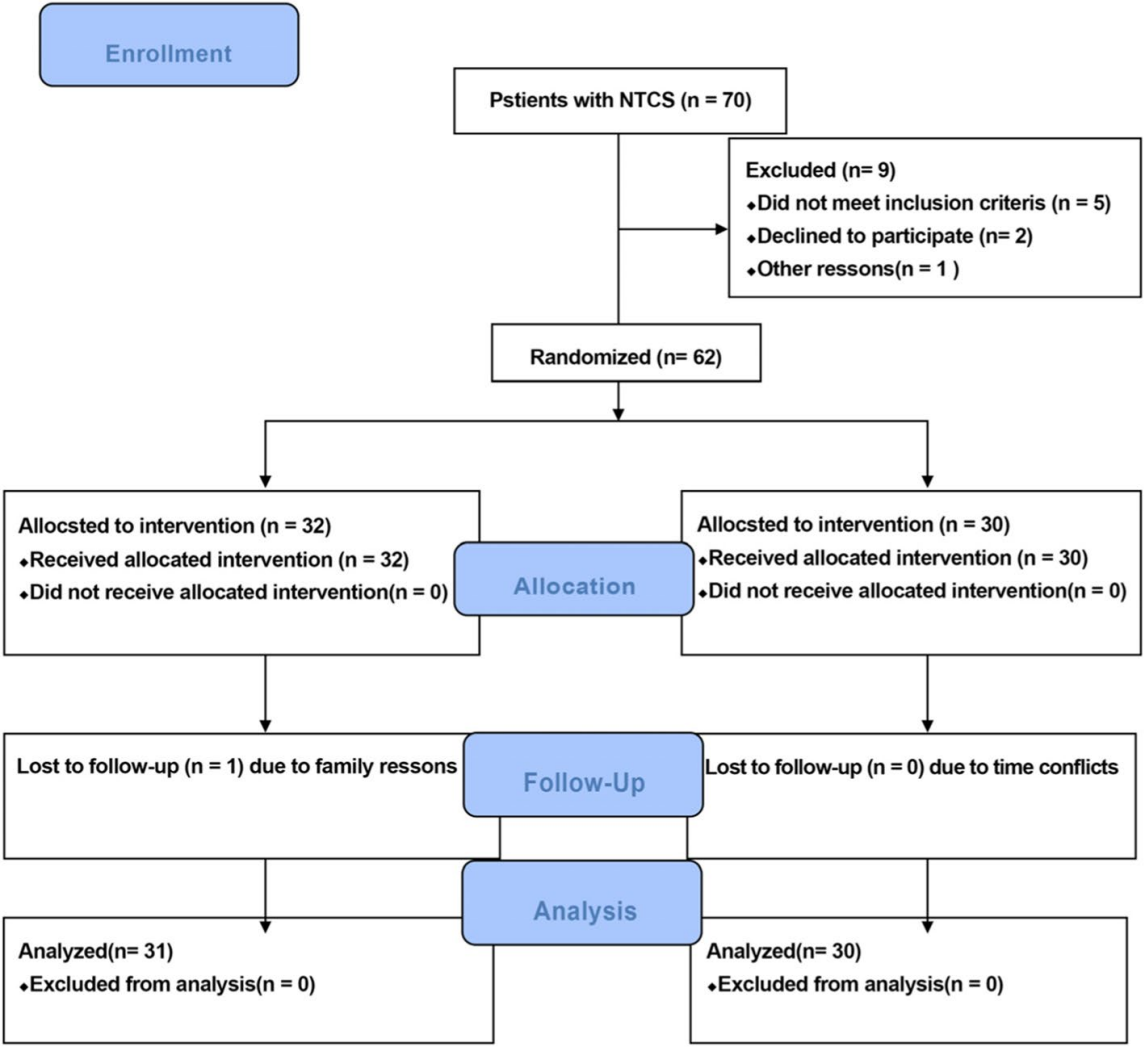
Participant recruitment schedule. Out of 70 participants recruited from the accessible population, 62 patients met the study selection criteria

The inclusion criteria were adults aged between 18 to 65 years with changes in cervical curvature showed by X-ray film inspection (such as straightening or anti-bow), and symptoms on the Clinical, Etiological, Anatomical, and Pathophysiological scale. Exclusion criteria were as follows:1) serious heart disease; 2) abnormal functions of the liver, brain, kidney, and other vital organ; 3) blood system diseases and serious mental illness; 5) cervical vertebrae fracture, dislocation, tuberculosis, serious infection; 6) contraindications for KT technique, including thrombosis, wounds, presence of cancer, intolerability of or allergy to surgical tape; 7) pregnancy. Out of 70 participants, 62 patients were recruited in this study (Fig. 1).

This study was registered on Chinese Clinical Trial Registry (http://www.chictr.org.cn) and the registration number was ChiCTR1900024907with the first registration on 03/08/2019(ChiCTR1900024907) . This study was approved by the local Ethics Committee of Wuhan Hospital of Traditional Chinese and Western Medicine (China)( [2016]3). All participants provided informed consent before participation.

### Study design

We performed a controlled clinical trial between March 2016 and December 2019. Patients were randomly assigned to KT group (*n* = 31) and control group (*n* = 30) by a random number table. Both groups were treated with physical modality therapy and multi-angle isometric resistance training for cervical muscles. The patients in the KT group were supplemented with Kinesio taping. All treatment interventions were carried out by a KT instructor with extensive experience, and he was blinded to the grouping information and measurements for outcomes, and the physical therapists were asked not to reveal the outcome message to the team members.

#### Kinesio taping application

**Fig. 2:**
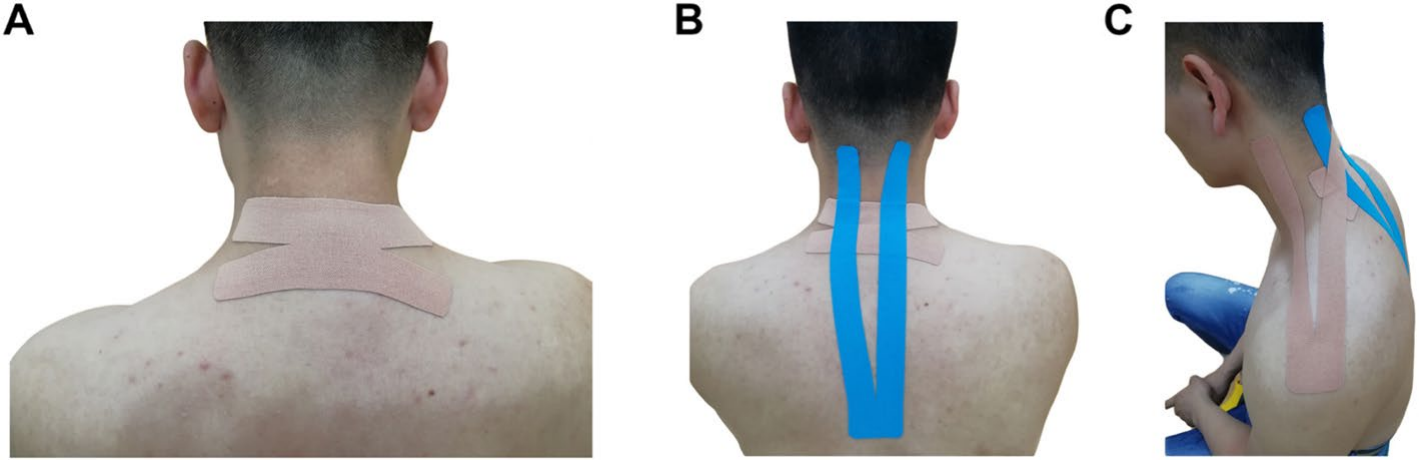
Application method of Kinesio Taping. **A**. The first layer of tape consisted of a X-strip placed over the pain spot, over the mid-cervical region (C3-C6), with the patient’s cervical spine in flexion to apply tension to the posterior structures. **B**. The overlying strip was a blue Y-strip placed perpendicular to the X-strip placed over the posterior cervical extension muscles, from the insertion to the origin, with paper-off tension, which the manufacturer applies to the tape against its paper backing at approximately 15% to 25% stretch. Each tail of the second strip (blue Y-strip, 2-tailed) was applied with the patient’s neck in a position of cervical contralateral side bending and rotation. **C**. The tape was placed from the dorsal region (T2-T5) to the upper-cervical region (C1-C2). The other strip was a Y-typed placed from acromion to the upper cervical region (C1-C2)

The tape (Kinesio Tex; Kinesio Nan Jing) used in this study was waterproof, porous, and adhesive. The tape with a width of 5 cm and a thickness of 0.5 mm was applied in the KT group. Patients received the following Kinesio Taping when seated. The first layer of the tape consisted of an X-strip placed over the trigger spot, over the mid-cervical region (C3-C6), with the patient’s cervical spine in flexion to apply tension to the posterior structures (Fig. 2A). The overlying strip was a blue Y-strip placed perpendicular to the X-strip placed over the posterior cervical extension muscles, from the insertion to the origin, with paper-off tension, which the manufacturer applies to the tape against its paper backing at approximately 15% to 25% stretch. Each tail of the second strip (blue Y-strip, 2-tailed) was applied with the patient’s neck in a position of cervical contralateral side bending and rotation (Fig. 2B). The tape was placed from the dorsal region (T2-T5) to the upper-cervical region (C1-C2). The other strip was a Y-typed placed from acromion to the upper cervical region (C1-C2) (Fig. 2C). Patients wore the Kinesio Tape 2 times a week during a 3-week period, and it was removed just before outcome assessment.

#### Manipulation interventions

The two groups received multi-angle isometric training for cervical muscles. The patients were trained by a physiotherapist. The specific trainings were as follows: 1) Cervical flexion training, the patient put his hands on the forehead to prevent head flexion action, and try to maintain balance so that the head does not move. 2) Cervical extension training. The patient's hands were overlapped and placed at the back of the head to prevent the head extended, while maintain the balance so that the head does not move. 3)Cervical rotation training. The patient's hands were placed on the one side of the head, with one hand exerting resistance to prevent the head to see the shoulder on the other side and maintained the balance. 4) Cervical lateral flexion training. The patient's hands were placed on the one side of the head, with one hand exerting resistance and mintained balance. 5) Cervical neutral position training. The palm of the hand applied external force from different directions (the forehead, the back pillow, and the bilateral cheeks), while the head and neck performed corresponding confrontation exercise to maintain the head in a neutral position. The above exercises were performed 30 min once time, 3 times per day, and 10 days for one course dur period.

### Evaluation methods

Four parameters were measured before the treatment and 3 weeks later. 1) Degree of pain was assessed by means of a 10-cm visual analog scale (VAS) from 0 (no pain) to 10 (unbearable pain). 2) Cervical vertebra function was evaluated by the neck disability index (NDI) [16], which measures symptoms and disability related to the neck. NDI could evaluate the following 10 items: the level of pain, daily life, extraction, reading, headache, concentration, work, driving and sleep. Each item was evaluated by 6 questions scoring 0–5 or 6. The total score of NDI was 50. And the score positively correlated with the neck dysfunction of the neck. 3) The range of motion for cervical vertebra was assessed (including flexion, extension, rotation, and lateral flexion). 4) The trapezius muscle state was evaluated with stiffness by Myoton hand-held dynamometer.

### Statistical analysis

Descriptive data are expressed as the mean ± SD. The VAS, NDI, CROM and muscle stiffness were assessed for the two groups at baseline and after treatment. Within—and between – group comparisons were performed using Student’s paired *t*-tests. The data were analyzed with SPSS Version 20.0 (SPSS Inc, USA), and statistical significance was set at *p* < 0.05.

## Results

Sixty-one participants (mean ± SD age, 42.5 ± 3.2 years; 36.07% female) satisfied the eligibility criteria, and were randomized to the KT group (*n* = 31) or control group (*n* = 30). There was no significant difference between KT group and control group in VAS (F = 1.902, *P* = 0.216), NDI scores (F = 0.244, *P* = 0.15) and CROM before treatment (*p* > 0.05, Table 1).

**Table 1:** Baseline demographics for both groups before treatment

	KT group	Control group	*P* value
Gender(Male/Femle) n	18/13	21/9	0.523
Age (years)	43.4 ± 2.4	41.3 ± 1.9	0.324
Neck pain	7.05 ± 0.42	6.97 ± 0.56	0.216
Neck Disability Index	38.41 ± 4.38	37.64 ± 5.47	0.15
Cervical range of motion			
Flexion	22.31 ± 2.56	22.56 ± 2.75	0.836
Extension	16.43 ± 1.34	17.96 ± 2.53	0.478
Left lateral flexion	23.45 ± 3.34	22.73 ± 4.21	0.942
Right lateral flexion	19.42 ± 4.33	21.91 ± 3.84	0.285
Left rotation	24.37 ± 3.46	26.66 ± 4.29	0.437
Right rotation	25.60 ± 3.87	26.45 ± 2.76	0.213

Both interventions were performed 2 times a week during the 3-week period by the random number table method. The data of the two groups (Table 2) were compared, and there was no statistically significant difference between the two groups (*P* > 0.05).

**Table 2:** Comparison of VAS, DNI and Cervical range of motionbetween two groups

	**KT group**		**Control group**	
	before treatment	3w posttreatment	before treatment	3w posttreatment
VAS	7.05 ± 0.42	3.75 ± 0.83^△^	6.92 ± 0.567	4.63 ± 1.39
NDI	38.41 ± 4.38	24.16 ± 3.42^#*^	37.61 ± 5.47	28.05 ± 3.67^#^
Flexion	22.25 ± 2.56	36.61 ± 3.24^#*^	22.76 ± 2.75	31.68 ± 1.23^#^
Extension	16.42 ± 1.34	31.42 ± 3.45^#*^	17.76 ± 2.53	25.49 ± 4.17^#^
Left lateral flexion	23.45 ± 3.34	32.12 ± 4.34 ^#△^	22.73 ± 4.21	30.19 ± 4.12^#^
Right lateral flexion	19.42 ± 4.33	27.67 ± 5.32^#△^	21.91 ± 3.84	28.68 ± 4.46^#^
Left rotation	24.35 ± 3.46	42.38 ± 2.45^#△^	26.47 ± 4.29	39.57 ± 3.63^#^
Right rotation	25.60 ± 3.87	40.23 ± 3.89^#△^	26.45 ± 2.76	39.02 ± 4.51^#^

Values are mean ± SD

Compared within the same group after treatment, ^#^*P* < 0.05

Compared between goups after treatment

^*^P < 0.05

^△^*P* > 0.05

The interaction of group and time for the 2-by-2 mixed-model analysis of variance was statistically significant for NDI, flexion and extension. The patients who received the Kinesio Taping experienced a greater increase in cervical flexion and extension range of motion than the control group (P < 0.05). There was no significant interaction for VAS, CROM for right and left rotation, and right and left lateral flexion between groups (Table 2).

There was no significant difference between the two groups before treatment on muscle stiffness (*p* > 0.05). After the 3 weeks treatment period, the muscle stiffness in KT group was significantly lower than that in the control group (*p* < 0.05) ( Table 3).

**Table 3:** Comparison of muscle stiffness between two groups

	before treatment	3w post treatment	*P* value^△^
KT group	269.24 ± 35.18	221.37 ± 37.54	0.00
Control group	265.74 ± 24.39	239.16 ± 23.56	0.00
P value^*^	0.914	0.035	

Values are mean ± SD

^*^the difference between groups

^△^the difference within group

## Discussion

The results of this study suggested that the application of Kinesio Taping and multi-angle isometric resistance training had similar effects for pain reduction. Additionally, patients in both groups gained similar improvements in cervical extension, flexion, and lateral flexion in both directions. However, individuals who received the combination treatment of Kinesio Taping and multi-angle isometric resistance training exhibited a greater increase in cervical flexion and extension range of motion than those in the control group. The pain relief in neck for both groups were not statistically significant.

In routine practice, relieving muscle pain and regulating muscle tension was the key to treat patients with cervical spondylosis. Therefore, a variety of techniques were applied to perform the goals, such as biofeedback, manual therapy, Kinesio therapy, botulinum toxin, platelet rich plasma, and pulsed radiofrequency.

Biofeedback was performed by placing surface electrodes on the skin to measure the frequency, intensity and duration of muscle contractions in order to decrease the tension of cramped muscles or increase the activity of weak muscles [17]. Meanwhile, botulinum toxin mainly inhibited the release of inflammatory transmitters from sensory nerve endings, while it could achieve flaccidity by inhibiting the release of acetylcholine from nerve endings [18]. Platelet rich plasma (PRP) was obtained by centrifugation of autologous blood. Clinical injections of PRP in excess of physiological concentrations to the lesion could release a variety of bioactive proteins that inhibited the inflammatory response, and promoted tissue regeneration and healing [19]. As an emerging interventional therapy, pulsed radiofrequency had the advantages of accurate localization, reproducibility, high safety and no nerve damage. Currently, pulsed radiofrequency was widely used for joint pain [20].

Previous studies had reported that cervical spine thrust manipulation is effective for reducing pain in individuals with mechanical neck pain [21, 22]. Meanwhile, Kinesio Taping could improve pain-free shoulder range of motion but had no effect on spontaneous pain or function [23]. This study also demonstrated that the combination of Kinesio Taping and multi-angle isometric resistance training can reduce self-reported disability, which was measured by the NDI over the 3-week duration in the study. However, changes observed were lower than the reported MCID of 7 points for the NDI [24]. It is possible that consecutive applications of Kinesio Taping or cervical manipulation would result in greater changes.

Historically, the mechanisms of spinal thrust manipulation have been primarily assumed to be biomechanical, but recently it has been purported that the mechanisms may be neurophysiological [25]. It is also possible that spinal thrust manipulation may result in a decrease in thermal pain sensitivity.

Previous study have confirmed that isometric contraction training is significantly better than isotonic in patients with NTCS [26]. Lindsay reported the training of isometric tetanic contractions could improve contractile function of dystrophin-deficient muscle, indicating a potential role for enhancing muscle strength in patients with DMD and BMD [27]. Moreover, patients with poor performance after isotonic contraction treatment can still achieve better performance after giving equal length contraction training. This study also showed that patients received either intervention could exhibit subtle increase in CROM. This was in agreement with findings from previous studies, which showed an improvement in movement and pain relief after applying the Kinesio Taping [28–32]. In a previous study, it displayed that mobilization technique with KT improved the ROM of the trapeziometacarpal joint [33]. Another study also reported improvement in stiffness after using Kinesio taping in patients with first metacarpal joint osteoarthritis [34]. Similar to aforementioned findings, some studies have suggested KT, as an adjunctive intervention, cpuld improve the ROM in rheumatoid hand. The changes were significantly greater in cervical flexion and extension range of motion than those in the control group, but the differences of other CROM were subtle. It is possible that greater changes in CROM could be observed from multiple applications of each intervention over a longer period.

However, there were some limitations in this study that should be noted. First, for some of the comparisons, the sample size was insufficient to demonstrate statistical significance for the clinical improvement observed, and further studies should therefore aim to calculate the sample size with sufficient power with a higher homogeneity in recruited participants from multiple centers. Second, we did not perform long-term follow-up evaluations to assess those changes. Further studies could implement longer follow-up periods to investigate the exact influence of KT on patients with NTCS.

## Conclusion

Patients with cervical spondylosis receiving an application of Kinesio Taping exhibited statistically significant improvements in pain levels and range of cervical motion immediately following application of the Kinesio Taping and after 3-weeks follow-up period. However, the improvements were subtle and may not be clinically meaningful. Further studies should investigate whether Kinesio Taping provides optimized outcomes when added to physical therapy interventions with proven efficacy.

## Data Availability

The datasets used and/or analyzed during the current study available from the corresponding author on reasonable request.
